# How to Transform a Perforator Propeller Flap into a Keystone Flap in Case of Unsatisfying Perforator Vessel Local Perforator Flap Coverage in Limbs

**DOI:** 10.1055/s-0042-1744416

**Published:** 2023-03-28

**Authors:** Elena Ciucur, Hadj Boukhenouna, Benjamin Guena, I. Garrido-Stowhas, Christian Herlin, Benoit Chaput

**Affiliations:** 1 Department of Plastic Reconstructive Surgery and Burns, Lapeyronie University Hospital, Montpellier, France; 2 Department of Plastic Reconstructive Surgery and Burns, Rangueil University Hospital, Toulouse, France

**Keywords:** propeller flap, intraoperative transformation, upper extremity, lower extremity, reconstruction

## Abstract

Moderate soft-tissue defects need stable coverage, ideally with tissue of similar characteristics and low donor site morbidity. We propose a simple technique for the coverage of moderate skin defects in the limbs. It allows intraoperative transformation of a propeller perforator flap (PPF) into a keystone design perforator flap (KDPF) in cases of unsatisfying perforator vessel or in cases of unpredictable intraoperative events. Between March 2013 and July 2019, nine patients with moderate soft-tissue defects (mean defect size 4.5 × 7.6 cm) in the limbs (two on the upper limbs and seven on the lower limbs) were covered using this technique. We performed four PPFs and five KDPFs. The mean follow-up was 5 months. There was one complication, partial distal tip necrosis in a PPF located in the leg, which healed by secondary intention within 3 weeks. The donor site was closed directly in all cases. No functional impairments were noted regardless of the perforator flap utilized. This technique enables us to employ flexible surgical strategies and allows us to make adjustments based on the patient's vascular anatomy.

## Introduction

Regardless of etiology, in case of moderate cutaneous defects of the limbs, a stable coverage is mandatory, ideally with tissue of similar characteristics and low donor site morbidity.

Perforator flaps serve all these purposes well for both traumatic and nontraumatic defects.

The propeller perforator flap (PPF) is nowadays in common use, despite the fact that good postoperative results rely on several factors, including a good understanding of the local anatomy, satisfactory local blood flow, and a technically challenging dissection. Complication rates (18.2–25.2% 1 2 ) are acceptable once beyond the learning curve.

In 2003, Behan 3 first described the keystone design perforator flap (KDPF) to cover defects following skin cancer excision. Since then, the KDPF proved to be a robust and easy-to-harvest flap.

We propose a simple intraoperative transformation for the coverage of moderate skin defects in the limbs. It allows intraoperative transformation of a PPF into a KDPF where there are insufficient perforator vessels. This also provides an alternative plan in cases of unpredictable intraoperative events.

## Case

### Patients and Methods

Between March 2013 and July 2019, we used this technique for closure of nine moderate defects in nine patients (four men and five women). The main etiology was skin cancer (six melanomas, two Merkel cell carcinomas) and one nevus. Two defects were on the upper limbs and seven were on the lower limbs. The mean age was 64 years old and the mean defect size was 4.5 × 7.6 cm.

### Patient Characteristics

Patient characteristics are presented in Table 1 .

**Table 1:** Clinical data

Patient no./sex	Age	Etiology	Smoking	Past medical history
**1/M**	80	Melanoma	No	Diabetes Ischemic heart disease
**2/F**	58	Melanoma	No	No
**3/F**	54	Melanoma	Yes	No
**4/F**	41	Nevi	No	No
**5/F**	83	Merkel cell carcinoma	No	Ischemic heart disease
**6/M**	82	Melanoma	No	Ischemic heart disease
**7/M**	67	Melanoma	No	Essential thrombocythemia
**8/F**	45	Melanoma	Yes	No
**9/M**	69	Merkel cell carcinoma	No	No

All patients with melanoma or Merkel cell carcinoma had benefitted from an initial excisional biopsy of the lesion for diagnostic purposes, and in all cases, the margins were clear. A wide excision within 2 cm associated with a sentinel lymph node biopsy (SLNB) was decided in accordance with national guidelines. For one patient who had a nevus excision, a 5-mm-margin excision was done. In all cases, moderate defects without the possibility of direct closure were present.

The study was approved by the Institutional Review Board of the Plastic Surgery Toulouse University Hospital (Toulouse, Rangueil, France; Number: 10.2018.02). All patients gave oral and written consent.

### Surgical Technique

All procedures were performed under general anesthesia and blue dye (2-mL *bleu patenté* V Guerbet 2.5%) was injected intraoperatively at the site of the initial scar, to help with the SLN identification. No tourniquet was utilized. The resection was designed in an elliptical fashion.

A perforator vessel was identified in close proximity of one end of the soft-tissue defect with the help of an acoustic Doppler (8 MHz, HADECO ES-100VX, Lemoine Medical, Entraigues, France). At the site of maximum skin laxity, a PPF was designed, parallel to the vascular axis of the limb. To increase the specificity of the acoustic Doppler, we performed the Mun and Jeon test. 4 A KDPF was designed on the same side of maximum skin laxity.

Once the resection was done, we described two possible situations.

#### Type 1 Technique

**Fig. 1:**
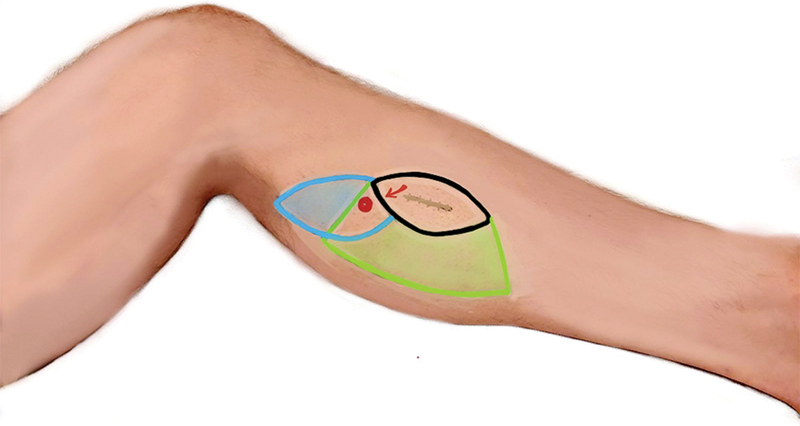
Type 1 technique. The propeller perforator flap (PPF) is indicated in *blue* ; the keystone design perforator flap (KDPF) in *green* . The *red arrow* indicates the exploratory pathway.

In the first case, the exploration of the perforator vessel is made by a subcutaneous dissection through the defect margins without making use of a complementary incision ( Fig. 1 ).

Once identified, if the perforator vessel is deemed satisfactory (in caliber and pulsatility), the PPF is harvested without the fascia. In case of a PPF, the fascia can be incised in the proximity of the perforator vessel to ease the rotation and the inset of the flap. If the perforator is unsatisfactory, the dissection is directed toward the KDPF and the fascia can be incised to facilitate the advancement of the flap.

An important technical point is not to undermine more than 50% of the surface of the KDPF while exploring the perforator vessels.

#### Type 2 Technique

**Fig. 2:**
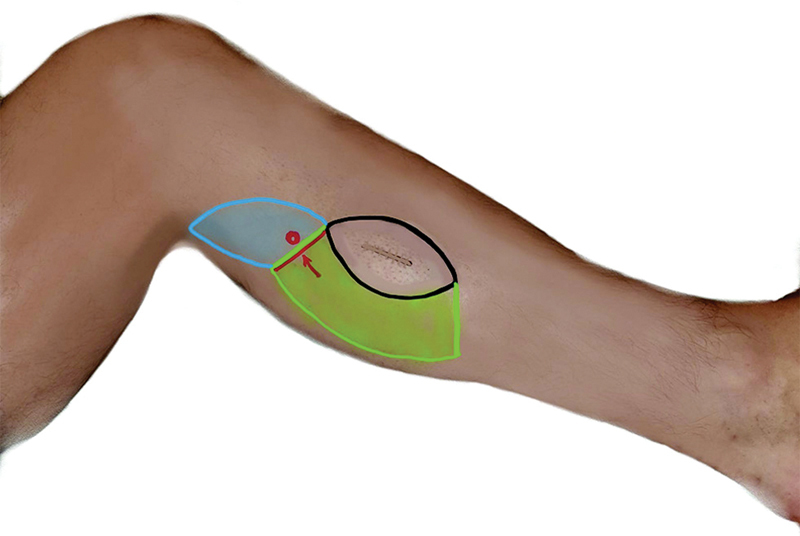
Type 2 technique. The propeller perforator flap (PPF) is indicated in *blue* ; the keystone design perforator flap (KDPF) in *green* . The *red line* indicates the exploratory pathway.

In the second scenario, the perforator vessel is not accessible to dissection through the defect's margins and thus requires a complementary incision ( Fig. 2 ).

In this situation, an exploratory incision on the border of the KDPF closest to the perforator vessel is made to inspect it. Depending on the quality of the perforator vessel, we orient our dissection to a either the PPF or a KDPF.

**Fig. 3:**
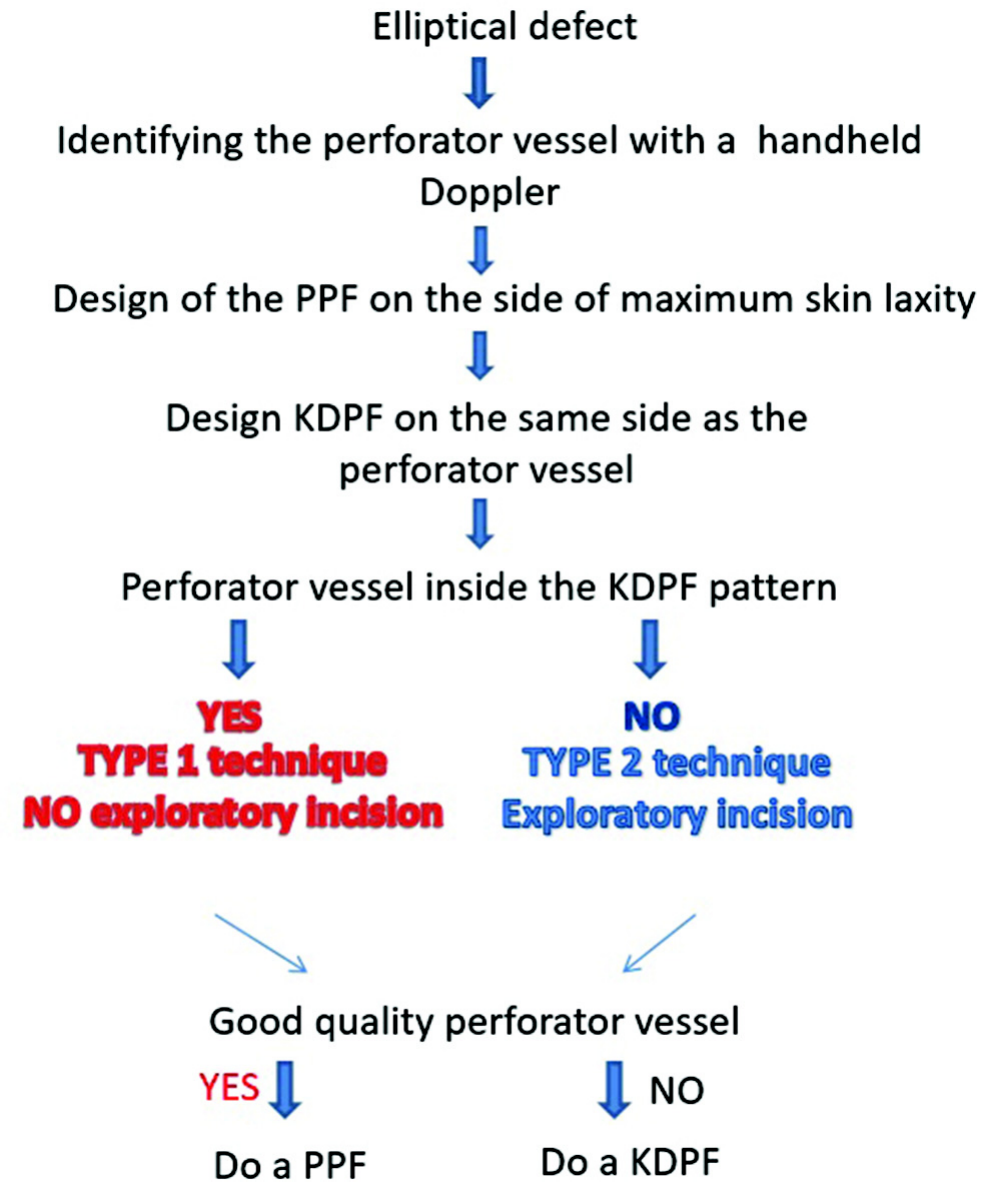
The decisional algorithm between the two types of techniques.

The decisional algorithm between type 1 and 2 technique is described in Fig. 3 .

### Results

In our series, we performed four PPFs, and in five cases, a safe intraoperative conversion into a KDPF type 2a was performed. The average flap size was 4.8 × 10.4 cm. The characteristics of the flaps and the defects are described in Table 2 . No particular observation of the flap was performed in the immediate postoperative period. No immobilization was used. The limb was placed in an elevated position, and we advised the patients to avoid physical exercise for 15 days postsurgery. A course of low molecular weight heparin (LWMH) was prescribed for 15 days to prevent deep vein thrombosis.

**Table 2:** Summary of flap characteristics

Patient no.	Site	Defect dimensions (cm)	Flap type/angle of rotation/vessel source	Flap dimensions (cm)	Harvesting plane	Donor site	Operative time (min)	Complications	Follow-up (mo)
**1**	Lateral upper thigh	5 × 10	PPF/90 degrees LCFA	5 × 9	Above the fascia	Direct closure	132	No	8
**2**	Lateral proximal third leg	5 × 8	PPF/180 degrees TAPF	5 × 9	Above the fascia	Direct closure	91	No	6
**3**	Lateral distal third leg	4.5 × 9	PPF/180 degrees PAPF	4.5 × 9	Under the fascia	Direct closure	75	Distal tip necrosis	8
**4**	Medial middle third leg	3 × 5	KDPF type 2a TPAPF	3.5 × 7	Above the fascia	Direct closure	30	No	3
**5**	Medial proximal third leg	7 × 9	KDPF type 2a TPAPF	8 × 13	Above the fascia	Direct closure	62	No	3
**6**	Lateral middle third arm	5 × 5	KDPF type 2a RCAPF	6 × 15	Above the fascia	Direct closure	50	No	6
**7**	Medial distal third leg	3.5 × 9	KDPF type 2a TPAPF	4 × 13	Above the fascia	Direct closure		No	6
**8**	Lateral knee	4 × 7	KDPF type 2a SLGAPF	4 × 10	Above the fascia	Direct closure	45	No	6
**9**	Lateral proximal forearm	4 × 7	PPF/90 degrees PIAPF	4 × 9	Under the fascia	Direct closure	63	No	3

Abbreviations: KDPF, keystone design perforator flap; LCFAPF, lateral circumflex femoral artery perforator flap; PAPF, peroneal artery perforator flap; PIAPF, posterior interosseous artery perforator flap; PPF, propeller perforator flap; PTAPF, posterior tibial artery perforator flap; RCAPF, radial collateral artery perforator flap; SLGAPF, superior lateral genicular artery perforator flap; TAPF, tibial anterior perforator artery flap.

We had one complication, partial necrosis of the distal tip of the PPF, in one patient who was a smoker. The flap was located on the leg and the defect after the necrosectomy at postoperative day 3. After the necrosectomy, the patient was discharged, and the defect healed by secondary intention within 3 weeks. In seven cases (two PPF flaps and five KDPF), surgery was performed in an outpatient setting. The follow-up averaged 5 months. We did not note any functional impairment, and the patients were satisfied with the aesthetic results.

#### Case 1

**Fig. 4:**
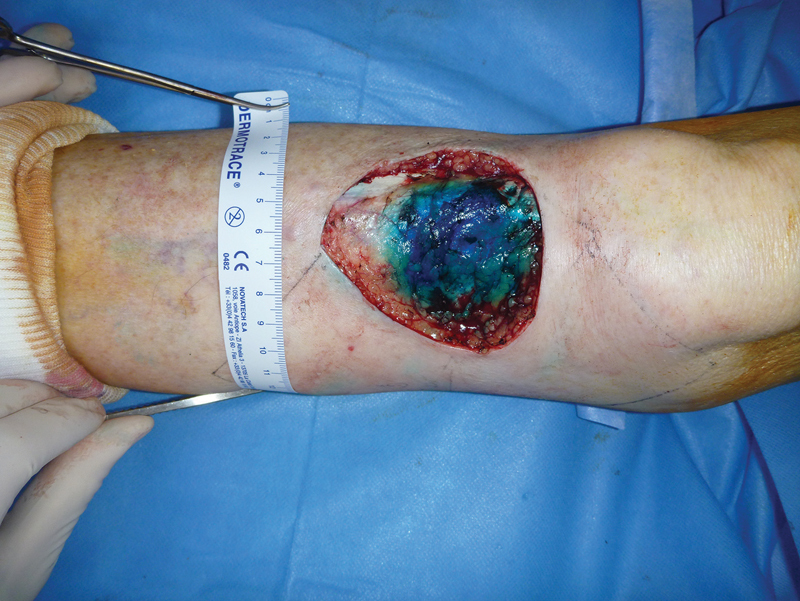
Soft-tissue defect in the anterior proximal leg (size 4 × 7 cm) after melanoma excision.

**Fig. 5:**
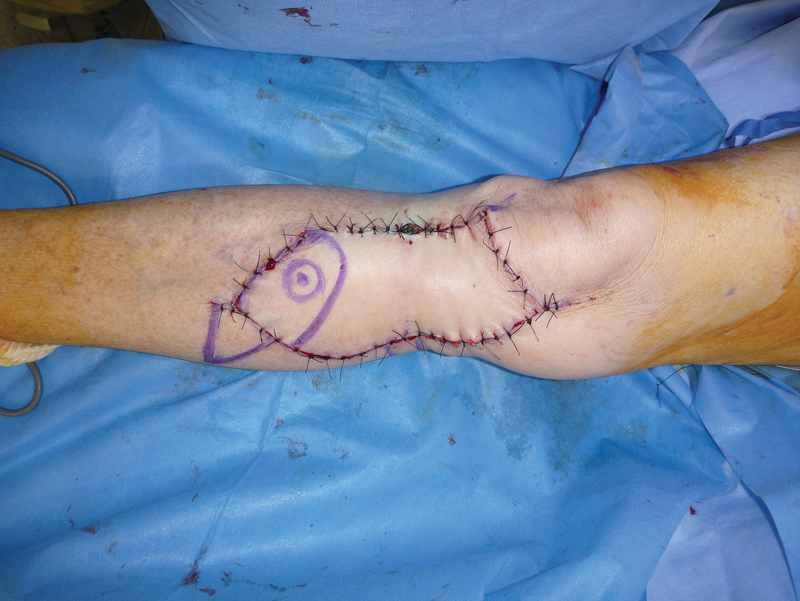
Due to unsatisfactory perforator vessel, a Keystone flap was done.

**Fig. 6:**
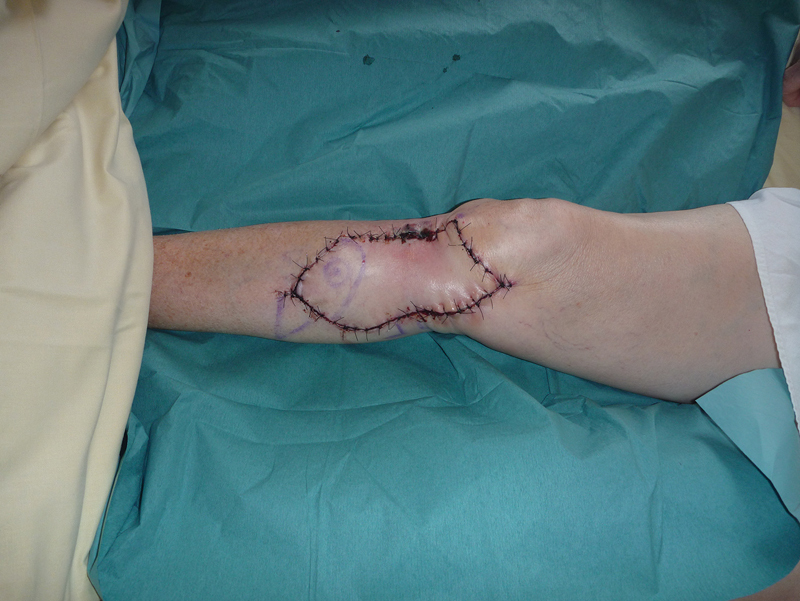
Result at 3 weeks postsurgery.

The first case was an 80-year-old man with a defect after wide excision of a melanoma scar on the lateral proximal thigh. We performed a type 2 technique ( Figs. 4 , 5 and 6 ).

#### Case 2

**Fig. 7:**
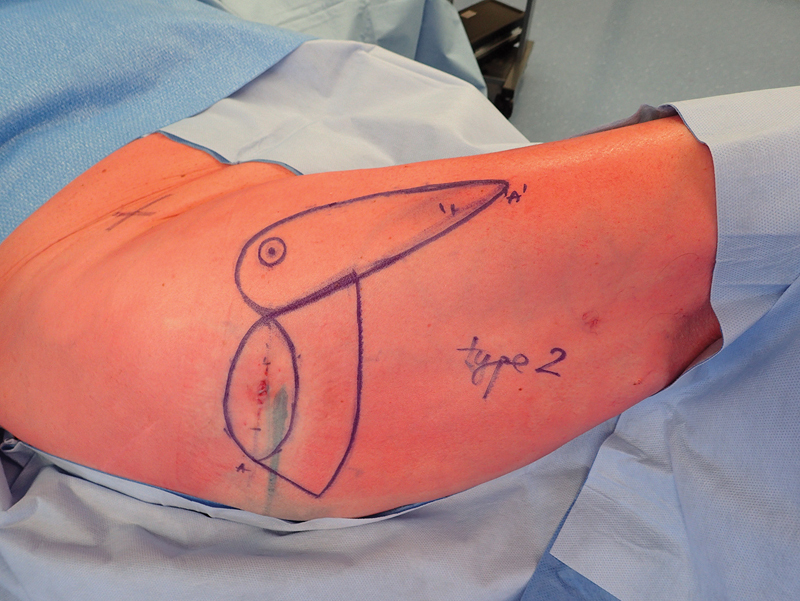
Presurgical markings (defect size 5 × 10 cm) on the lateral aspect of the thigh. A satisfactory perforator was located and a propeller perforator flap (PPF) was harvested (type 2 technique).

**Fig. 8:**
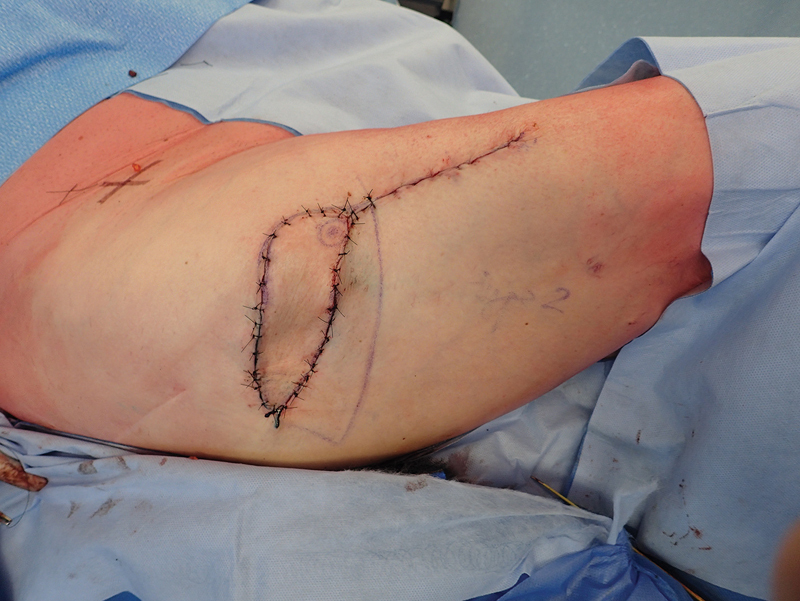
Final outcome.

The second case is an 85-year-old male, with a defect after wide excision of a Merkel cell carcinoma scar on the anterior proximal leg. A keystone flap was used ( Figs. 7 and 8 ).

## Discussion

Multiple etiologies can be considered in cases of moderate soft-tissue defects. The most frequent ones are trauma and after tumor excision.

When direct closure is not an option, the use of local free style perforators flaps allows more freedom in reconstructive surgery and the myriad choices available increase the safety of coverage. In our institution, in case of moderate cutaneous defects we tend to use the propeller local flaps. In our nine cases, the main indication was a local PPF. Two main advantages of the PPF flap are the broad mobilization options and a fill-in effect with minimal deformation at the donor site. The fill-in effect is very important because this allows tension-free closure, thereby avoiding dehiscence (one of the most common complications of KDPF 5 ).

Described in 1991, 6 the PPF is an island fasciocutaneous perforator flap capable of a rotation of 180 degrees, 7 harvested in the majority of cases from a single perforator vessel. It requires a more demanding dissection to correctly liberate the perforator vessels. Therefore, it is more prone to venous and arterial insufficiency, being dependent on the dynamic perforasome, rotation angle, 1 and quality of the perforator vessel.

Despite the preoperative identification of the perforator vessels with a Doppler, it is necessary to reassess the perforator vessel intraoperatively for pulsation and caliber to assure a viable coverage. 8 Harvesting a PPF in cases of unsatisfying perforator vessel exposes us to a much higher complication rate.

The purpose of this intraoperative transformation was to find a surgical alternative that would allow us a safe and technically accessible bailout in the cases where we are confronted with a poor-quality perforator vessel.

In the perforator era, our interest turned to the keystone flap, a versatile and straightforward flap.

The KDPF is an advancement flap based on random fasciocutaneous or musculocutaneous perforators. Its mobilization capacities are due to skin elasticity and stretching capabilities. 9 10 The incision of the fascia can augment its mobility, albeit only slightly. 10 The KDPF is a reliable flap with complications varying from 0 11 12 to 19.6%. 13

The most common complications known in the case of KDPF are wound infection and dehiscence. 5 13 14 15 This is probably due to the intrinsic tension at the suture line and through the central region of the flap. One option to avoid dehiscence at the closure line is to allow the donor site healing by secondary intention.

In our series, in five patients we found perforator vessels that were deemed unsatisfying. The intraoperative transformation into a KDPF, using our algorithm, allowed us an uneventful coverage of the defect. Even if the closure tension was significantly more important in the case of the KDPF than in the PPF patients, no dehiscence was observed despite the direct closure at the donor site. In our series, we had only one partial necrosis of the distal portion of a PPF. The propeller flap was based on a perforator of the peroneal artery, and had a rotation angle of more than 150 degrees in a leg defect in a patient who was a smoker. In this case, we probably should have selected intraoperative transformation into a KDPF (complication rate of 19.6% 13 in the lower limbs) despite the satisfactory aspect of the perforator vessels, because the patient had cumulative, multiple risk factors for a PPF flap (smoking, rotational angle of more than 150°, and peroneal perforator vessel origin 16 ).

The learning curve in cases of a PPF that requires a rotational angle of more than 90 degrees is longer than in cases of KDPF. The intraoperative transformation of the PPF into a KDPF is a useful training model for young surgeons, because the transformation is possible even in cases of technical error during the exploration of the perforator vessel. The young surgeon must keep in mind that the transformation into a keystone flap is no longer possible once the propeller flap is incised on its entire surface and the harvest done.

Another important use of this intraoperative transformation is in cases of incorrectly oriented scars that need a wider excision. We had three cases where the initial scar was oriented transversely on the limbs rather than longitudinally. In these cases, a KDPF is still possible, but a PPF presents a more anatomical position of the scars and exerts less tension on the donor site.

Despite the fact that in our series we used this technique primarily in carcinologic cases, we are confident that it can be useful in all types of moderate tissues defects, regardless of etiology.

Nowadays, as a result of a better understanding of skin blood supply, flaps are used on a regular basis, with an acceptable complication rate. This technique proposes intraoperative transformation of a more elegant but more complication-prone flap, the PPF, into a more robust one, the KDPF. This enables us to employ flexible surgical strategies while guarding a readily available bailout plan and making adjustments based on the patient's vascular anatomy.
